# Characterization by Gender of Frailty Syndrome in Elderly People according to Frail Trait Scale and Fried Frailty Phenotype

**DOI:** 10.3390/jpm12050712

**Published:** 2022-04-29

**Authors:** Iván Palomo, Francisco García, Cecilia Albala, Sergio Wehinger, Manuel Fuentes, Marcelo Alarcón, Diego Arauna, Hector Montecino, Diego Mendez, Magdalena Sepúlveda, Peggy Fuica, Eduardo Fuentes

**Affiliations:** 1 Thrombosis Research Center, Medical Technology School, Faculty of Health Sciences, Interuniversity Center for Healthy Aging, Universidad de Talca, Talca 3460000, Chile; ipalomo@utalca.cl (I.P.); snunez@utalca.cl (S.W.); manuelfuentesgarciatm@gmail.com (M.F.); malarcon@utalca.cl (M.A.); darauna@utalca.cl (D.A.); hector.montecino@utalca.cl (H.M.); diego.mendez@utalca.cl (D.M.); magdalena.sepulveda@utalca.cl (M.S.); pfuica15@alumnos.utalca.cl (P.F.); 2 Department of Geriatric Medicine, Complejo Hospitalario de Toledo, 45007 Toledo, Spain; franjogarcia@me.com; 3 Unidad de Nutrición Pública, Instituto de Nutrición y Tecnología de los Alimentos, Interuniversity Center for Healthy Aging, Universidad de Chile, Santiago 8320000, Chile; calbala@uchile.cl

**Keywords:** older people, frailty, frail trait scale, fried phenotype, gender

## Abstract

Background: Frailty has emerged as one of the main geriatric syndromes to be prevented in order to improve quality of health and life in the elderly. In this sense, the characterization of this syndrome through reliable and feasible diagnostic tools for clinical use, such as the Frail Trait Scale 5 (FTS-5) and Frail Trait Scale 3 (FTS-3), represents the basis for this objective. Objectives: To characterize the frailty syndrome in a population of older adults using FTS-5, FTS-3, and Fried phenotype (FP) as frailty diagnostic tools. Design: Cross-sectional study. Participants: 300 adults ≥65 years recruited from different Family Health Centers and community groups of older people in Talca, Chile. Methods: The diagnosis of frailty was made according to FP, FTS-5, and FTS-3 tools. Data about sociodemographic characteristics and anthropometric measurements were collected by a clinical interview by a previously trained health professional. Results: A total prevalence of frailty according to the FP of 19.7% was observed; while in the group of women and men it was 21.4% and 15.0%, respectively. Concerning the FTS-5 tool, the total prevalence of frailty was 18%, while in the group of women and men was 18.0% and 17.5%, respectively. The FTS-3 tool shows a total prevalence of frailty of 23.3%, while in the group of women and men a prevalence of 22.7% and 25.0%, respectively. A significant difference is observed with respect to the presence of the Fried criteria of “weakness” (women: 21.4%, men: 38.8%) and “weight loss” (women: 16.8%, men: 7.5%; *p* < 0.05). A significant difference is observed concerning the average score of “Handgrip” criteria, “walking time”, and “Physical Activity Scale for the Elderly” (PASE) between the group of women and men. Frailty, diagnosed by FTS-3, is significantly associated with the risk factors of overweight (body mass index ≥ 25) (OR: 10.225, 95% CI: 1.297–80.617) and advanced age (age ≥ 75 years) (OR: 1.839, 95% CI: 1.040–3.250). Conclusion: The prevalence of frailty observed with the FTS-5 (18%) and FTS-3 (23.3%) tools are similar to the prevalence observed through the FP (19.7%) and those reported in other observational studies. Considering the similar prevalence of frailty diagnosed with the three tools, FTS-3 should be a valuable tool for the screening of frailty in the community.

## 1. Introduction

Currently, research on the aging process and its key determinants has become a very relevant topic, due to the accelerated aging of the world population [1,2,3]. This is observed in the fact that 12% of the world population is ≥60 years old, and forecasts for the mid-century indicate that this figure could reach up to 21.5% [4]. Likewise, during this same period, the age group of ≥80 years would increase from 1.7% to 4.5% [4]. The situation of the Chilean population is similar to the world panorama, with an increase in life expectancy at birth of 4.2 years per decade, reaching 79 years [4,5]. Based on the above, it is expected that Chileans older than ≥60 years old will increase from 15.7% to 32.9% by the year 2050, and the population older than ≥80 years old could reach 10.3% [4]. According to the Worldwide Health Organization (WHO) in its “Integrated care for older people” guide (ICOPE guide), the frailty syndrome is a key determinant regarding the state of dependency, presence of chronic diseases, and quality of life in older people [6,7]. The frailty syndrome is defined as a preventable and reversible clinical state, in which the ability of older people to cope with everyday stressors is compromised by an increase in vulnerability and the physiological deterioration of age [8]. Recent results show a prevalence of frailty in Chile slightly higher than 20%, which is comparable with other South-American countries [9,10]. In 2014, the creation of a new tool with 12 criteria for the diagnosis of frailty, the Frailty Trait Scale (FTS-12), was reported, which presented better predictive values for mortality and adverse health events compared to the FP [11]. The Frailty Trait Scale will evaluate seven dimensions (energy balance-nutrition, physical activity, nervous system, vascular system, strength, endurance, and gait speed) represented by 12 items [11]. In the year 2020, with the purpose of creating a more practical tool with the same diagnostic properties, the authors developed two shortened versions of the FTS-12, the FTS-5 (5 criteria) and FTS-3 (3 criteria) [12]. Because of the importance of implementing a reliable, workable, and clinically relevant frailty diagnostic method, the objective of this study is to characterize the frailty syndrome in a population of older adults in the Maule region (Chile), using the FTS-5, FTS-3, and FP tools.

## 2. Methods

*Participants and study design*. The study was designed as a cross-sectional case-control study, with a representative sample of older persons (men and women, ≥65 years old) randomly selected from different Family Health Centers and community groups of older people in Talca, Chile (*n* = 300). The inclusion criterion was adults 65 years old and older. The exclusion criteria were the presence of cancer, Parkinson’s disease, or vascular accident, both self-reported by the participant and by identification in the medical record, and older people who will not be able to walk or speak will also be excluded, and those who are on statin therapy [13]. The study obtained approval from the ethics committee of Universidad de Talca and the written informed consent of each participant. The sample (men and women, ≥65 years old) size calculation will be made considering the prevalence of frailty in older adults of 24.6% [9], with a confidence level of 95%, statistical power of 80%, and a percentage of loss of 20%, using the software GRANMO calculator. The proportions of women and men will be determined by the relative amount of the adult population over 65 years of age based on data from the National Socioeconomic Characterization Survey (CASEN, 2017).

*Data collection.* Data about sociodemographic characteristics (gender, age, years of education, and residence) was collected by conducting a clinical interview by a previously trained health professional (four professionals). The interview included measurements to collect information about body mass index (BMI) (weight and height) and waist circumference. Abdominal obesity was defined with waist circumference >102 cm in men and >88 cm in women (ATP III criteria) [9].

*Frailty diagnosis.* The diagnosis of frailty was made according to FP (five criteria: slowness, weakness, weight loss, exhaustion, and low physical activity) (≥3 components was considered frail), FTS-5 (score >25 was considered frail) and FTS-3 (score >15 was considered frail) tools. FTS-5 considers the evaluation of five dimensions: energetic balance and nutrition (BMI criteria), physical activity (PASE score), nervous system (Romberg test score), strength (grip strength dynamometry), and walk speed (walk speed in 3 m). FTS-3 considers the evaluation of three dimensions: energetic balance and nutrition (BMI criteria), physical activity (PASE criteria), and nervous system (Romberg test criteria) [14]. The information (response to survey questions and quantitative data from the frailty scales) was recorded through a form generated by Google Forms on electronic devices.

*Statistical analysis.* Statistical analyses were performed with GraphPad Prism 8. Continuous variables were expressed as mean ± standard deviation (SD) or median (95% confidence interval; CI). Categorical variables were expressed as percentages and a 95% confidence interval (95% CI). In the analysis of differences between groups, the chi-squared test with Yate’s correction was used to assess differences in proportions and the Student T-test or the Mann Whitney test, as appropriate, to assess differences in means or medians. For comparisons using T tests, the homogeneity of the variance (using Bartlett’s test and homoscedasticity plot) and the normality of each variable (Shapiro-Wilk test, K-S test or Anderson-Darling test) were checked. Continuous variables were grouped to create ordinal categorical (binary) variables. Logistic regression models were performed to analyze the association between frailty and studied variables, unadjusted, and adjusted by age and gender. For this, a binary logistic regression was performed, where the dependent variable was the state of frailty (1: frail, 0: non-frail) and the covariates were the presence or absence of different health factors (1: presence, 0: absence), using SPSS 15 software. From the linear equations obtained, the values of “Exponential value of B” (Exp(B)) and their 95% confidence interval were obtained to establish the odds ratio (OR) associated with each variable. The significance of the association was obtained by means of the “sigma value” of each variable of the equation. Statistical details of logistic regressions can be observed in Tables S1 and S2. The *p*-values lower than 0.05 were considered statistically significant.

## 3. Results

### 3.1. Characterization of the Cohort by Gender

The studied cohort was composed of 300 participants, of which 73.3% were women and 26.7% were men (Table 1). The mean age of the cohort was 74.3 years, and no significant difference in this value was observed between men (74.1 years) and women (75 years). Regarding BMI, the cohort presented an average value of 29.7 kg/m2, and no significant difference was observed between women (29.7 kg/m^2^) and men (29.6 kg/m^2^). Likewise, the average value of years of education in the cohort was 9.4 years, and no significant difference was observed between the group of women (9.3 years) and men (9.6 years). On the other hand, a higher prevalence of abdominal obesity is observed in older women (73.6%) compared to the group of older men (48.8%), also presenting a prevalence of 28% in the entire cohort. Similarly, there is also a higher prevalence of “living alone” in the group of older women compared to the group of older men, observing a prevalence of 28% in the entire cohort.

**Figure 1:**
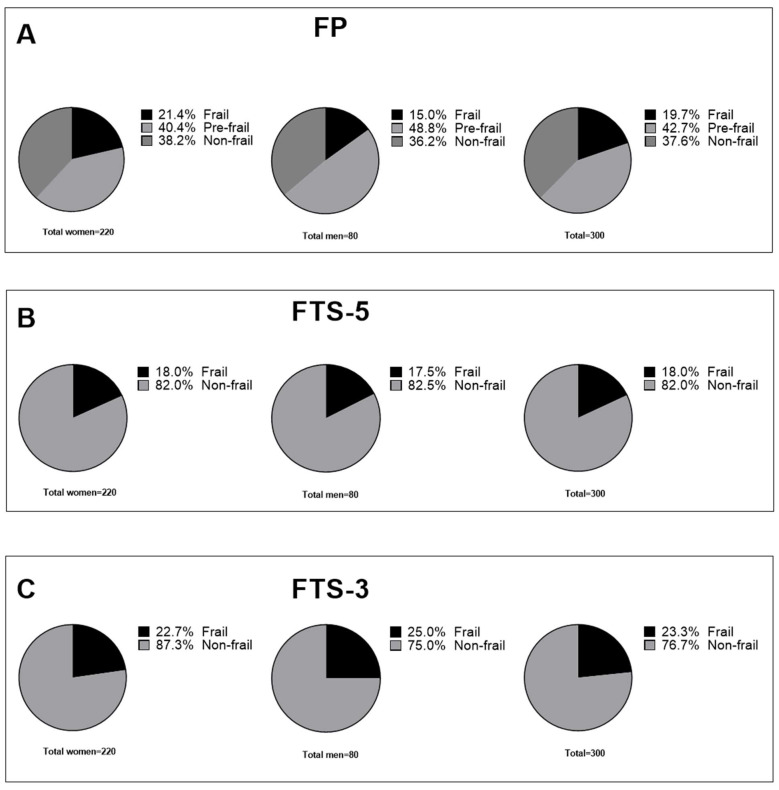
Distribution of frailty syndrome by FP, FTS-5, and FTS-3. (**A**) Distribution of frailty syndrome in women, men, and all participants, according to Fried phenotype. (**B**) Distribution of frailty syndrome in women, men, and all participants according to FTS-5. (**C**) Distribution of frailty syndrome in women, men, and all participants according to FTS-3. FTS-5, Frail Trait Scale 5; FTS-3, Frail Trait Scale 3; FP, Fried Phenotype.

Continuing with the description of the studied cohort, Figure 1 shows the distribution of the frailty syndrome according to the FP, FTS-5, and FTS-3 diagnostic tools. Figure 1A shows the distribution of the frailty syndrome according to the FP. This shows that the total prevalence of frailty was 19.7%, while in the group of women and men it was 21.4% and 15.0%, respectively. The prevalence of global pre-frailty was 42.7%, while in the group of women and men it was 40.4% and 48.8%, respectively. Figure 1B shows the distribution of the frailty syndrome according to the FTS-5 tool. A total prevalence of frailty of 18% is observed, while in the group of women and men a prevalence of 18.0% and 17.5%, respectively, is observed. In Figure 1C, this same distribution is observed according to the FTS-3 tool. In this, a total prevalence of frailty of 23.3% is observed, while in the group of women and men a prevalence of 22.7% and 25.0% are observed, respectively.

### 3.2. Comparison of Frailty Profiles by Gender

The frequency distributions of the five components of the FP, grouped by sex, are shown in Table 2. In this table, a significant difference is observed in the presence of the “weakness” criteria (women: 21.4%, men: 38.8%; *p* < 0.01) and “weight loss” (women: 16.8%, men: 7.5%; *p* < 0.05). However, this is not observed in the criteria of “slowness” (women: 25.0%, men: 17.5%), “exhaustion” (women: 30.9%, men: 25.0%) and “low physical activity” (women: 36.4 %, men: 33.8%). In this same sense, when comparing the prevalence according to the number of frailty components present, no significant difference is observed between the group of men and women of the cohort. Likewise, when comparing the total prevalence of frail (women: 21.4%, men: 15.0%) and pre-frail (women: 40.5%, men: 48.8%) between the group of men and women, no significant difference is observed either.

The average score of the five criteria of the FTS-5, grouped by gender, can be seen in Table 3. This shows a significant difference in the average score of the “Handgrip” criteria (2.1 ± 1.9 vs. 1.3 ± 1.7, *p* < 0.001), “walking time” (2.4 ± 2.0 vs. 1.8 ± 1.7, *p* < 0.01), and “PASE” (4.7 ± 2.3 vs. 3.8 ± 2.6, *p* < 0.001) between the group of women and men, respectively. Likewise, Table 3 shows the distribution of the organized cohort according to the assignment of quartiles of the FTS-5 score (Q1: more robust; Q4: more frail). There is no significant difference in this distribution when comparing the group of men and women. Likewise, when comparing the total prevalence of frailty estimated by the FTS-5 between the group of men and women (women: 21.4%, men: 15.0%) and FTS-3 (women: 22.7%, men: 25.0%), no significant difference is observed.

### 3.3. Association Studies between Frailty and Relevant Health Factors

The association study between the state of frailty diagnosed according to the FP and relevant health variables can be seen in Table 4. Considering the groups of frail and non-frail people, it is observed in model 1 that there is no significant association of frailty with the variables of sex (female) and age (age ≥ 75 years). In model 2, which adds the variables of years of education (Years of education ≤ 8), excess weight (BMI ≥ 25 kg/m^2^), and abdominal obesity (ATP III criteria), a significant association with education is observed (OR: 2.316, *p* < 0.05). Continuing with model 3, which adds the variables of “living alone” and “self-medication”, the significant association with the variable of years of education is maintained (OR: 2.240, *p* < 0.05). It should be noted that in both models 2 and 3, the variable BMI ≥ 25 kg/m^2^ presents a high OR (OR: 2.680 for model 1; OR: 2.063 for model 3), however, this association does not present significance. Considering the group of frail vs. pre-frail people, it can be observed in both models 1, 2, and 3 that there is no significant association with the aforementioned variables. However, it is observed that in both models 2 and 3, the variable BMI ≥ 25 kg/m^2^ presents a high OR (OR: 2.825 for model 1; OR: 3.191 for model 3), but without a statistically significant association.

On the other hand, the association study between the state of frailty diagnosed according to the FTS-5 and relevant health variables can be seen in Table 5. This logistic regression analysis considered the groups of frail vs. non-frail people. It can be observed in model 1, which included the variables of sex (woman) and age (age ≥ 75 years), that there is no association between these previously named variables. Model 2, in the same way as the previously described association study, adds the variables of years of education (Years of education ≤ 8), excess weight (BMI ≥ 25 kg/m^2^), and abdominal obesity (ATP III criteria). In this, a significant association is observed with the variable of “excess weight” (BMI ≥ 25 kg/m^2^) with an OR of 8.168 (*p* < 0.05). However, in model 3, which adds the variables of “living alone” and “self-medication”, the association with the variable of “excess weight” loses its significance.

The association study between the state of frailty diagnosed according to the FTS-3 and relevant health variables previously listed can be seen in Table 5. This logistic regression analysis considered the groups of frail vs. non-frail people. It can be observed in model 1, which included the variables of sex (female) and age (age ≥ 75 years), a significant association with the variable “age ≥ 75 years” (OR: 1.726, *p* < 0.05). Model 2 adds the variables of years of education (Years of education ≤ 8), excess weight (BMI ≥ 25), and abdominal obesity (ATP III criteria). In this, a significant association is observed with the variable “BMI ≥ 25 kg/m^2^” (OR: 10.381, *p* < 0.05), while the variable “age ≥ 75 years” maintains its significant association (OR: 1.804, *p* < 0.05). In model 3, which adds the variables of “living alone” and “self-medication”, the previously mentioned significant associations are maintained, observing an OR of 1.839 for the variable “age ≥ 75 years” (*p* < 0.05) and an OR of 10.225 (*p* < 0.05) for the variable “BMI ≥ 25 kg/m^2^”.

## 4. Discussion

The results of this study considered a cohort of 300 participants, with a greater presence of female participants, which is observed in our previous study when recruitment considers health care centers [9]. A relatively aged cohort (average age 74 years) with a high average BMI (29.7 kg/m^2^) and an average education of 9.4 years are observed. In agreement with the observations regarding the BMI parameter, a high prevalence of abdominal obesity was observed in the cohort, which was exacerbated in the group of older women. This characteristic has been strongly associated with frailty syndrome, and recently was reported that older adults that present with a high category of waist circumference had a pooled 57% higher risk of frailty than those with a normal waist circumference [15,16,17].

The main objective of this study was to characterize the frailty syndrome in older adults in the Maule Region, Chile, using the FTS-5 and FTS-3 diagnostic tools. These results were compared with the characterization obtained through the FP, which has been recognized as one of the standards of frailty tools [13,18,19]. In addition, this research sets the precedent of being the first characterization in a South American cohort of older people, using the FTS tools, which have been widely validated in European cohorts [11,14,20,21].

The results obtained regarding the FP indicate a total prevalence of frailty of 19.7%, being higher in women (21.4%) than in men (15%). The prevalence of pre-frailty was 42.7%. These results are similar to those obtained in our previous research (24.6%, diagnosis by FP) and those observed by Albala et al. (13.9%, diagnosis by FP) [9,22]. According to the observations in the characterization of the cohort according to the FP, the group of women presented with an increase in the criterion of “involuntary weight loss”, and to a lesser extent in the criterion of “slowness”, compared to the group of men. Involuntary weight loss is associated with considerable morbidity and mortality in older adults, while the criteria “slowness” has been well characterized as an estimator of the risk of frailty, as well as an estimator of mortality and hospitalization events [23,24]. Recently, it was reported that involuntary weight loss in older persons is associated with late-life depression, which also was associated with a higher risk of frailty and would explain the increased prevalence of this criteria in the female group due to depression being more prevalent in this group [25,26]. On the other hand, this group of men shows an increase in the “weakness” criterion (grip strength), compared to the group of women. Loss of strength has been closely linked to frailty, as well as other adverse health events [27,28]. The associated cause is an exacerbation of the sarcopenia process, which is associated with aging [27]. This is why frailty intervention and prevention programs consider the incorporation of low-load exercises to recover muscle mass and improve its quality [6,29,30].

Considering the results obtained using the FTS-5 tool, a total prevalence of 18% is observed, while a prevalence of 18.1% and 17.5% was observed for women and men, respectively. These results are comparable to those observed with the FP and are similar to those observed in other South American countries and slightly lower than other European countries such as Spain [10,21,31]. On the other hand, considering the results obtained with the FTS-3 tool, a total prevalence of frailty of 23.3% is observed, while in men and women a prevalence of 22.7% and 25%, respectively, is observed. These values are slightly higher than those observed with the FP and FTS-5; however, this is not significantly different to those obtained by the two tools named above. This fact was expected, due to the sensitivity and specificity reported about FTS-5 and FTS-3 tools [12].

The FTS-5 and FTS-3 criteria present a score range of 0–10, where a higher score represents a worse performance in that criterion [12]. In this context, the group of older women presented with a worse performance in the criteria of grip strength, walking speed in 3 m, and PASE. This indicates that the main domains affected in the group of older women would be strength, gait speed, and physical activity, while in the group of men the main domains affected would be the nervous system, physical activity, and nutritional status/energy balance [11]. From this, we infer that there are different population profiles of frailty according to sex, which is also supported by the fact that the aging process is different in men and women [32]. A similar fact has been observed in other studies in larger European cohorts, suggesting that frailty presents with different subtypes depending on the affected domains [33,34]. The distribution of quartiles of the FTS-5 score indicates the distribution of the population according to its level of frailty. These results indicate that a q homogeneous distribution between quartiles is observed (around 25% of the cohort in each quartile) according to sex. The majority of studies show that females are frailer than males, at all ages, when frailty is assessed mainly by the FP, despite that the female group presents at lower mortality risk [35]. These discrepancies are likely due to a combination of behavioral, social, and biological factors, have important consequences for frailty susceptibility, and suggest the need for a diagnostic tool of frailty that considers this fact [13]. An important limitation to consider in the comparisons according to sex carried out in this study, is the unequal size of men and women group. We observe this because of the effect of sampling carried out in health centers, being evidenced in a previous report and highlighting the need to corroborate the observed associations and differences in more extensive studies [9]. In this sense, it is necessary that future studies consider the statistical technique of boostrapping, to test the stability of the model and the sample distribution.

Association studies using the FP indicate a strong association between frailty and low schooling, which indicates that education could be a relevant factor in the prevention or intervention of frailty in this population, but the current evidence is lacking [36,37]. Low education in elderly people has been observed in cohorts from Brazil and Netherlands and has also been associated with frailty [36,38,39]. Recently, the importance of the “cognitive reserve” regarding frailty has been established, considering the variable of “years and education” as an intrinsic variable of this; however, due to the low standardization of the measurement of this reserve, more extensive studies are necessary to evaluate its incorporation into frailty models [40]. On the other hand, the health determinants used in the study do not present a significant association in the transition process from pre-frailty to frailty, with more extensive studies being necessary.

Association studies using the FTS-5 tool indicate a potential and strong association with the factor “BMI ≥ 25 kg/m^2^”; however, this factor loses significance in subsequent models. This observation could be clarified by an increase in the size of the cohort. A high BMI in older persons was related to frailty and is a good predictor of mortality; however, some studies indicate a controversial association between BMI and frailty, showing a U-shaped curve [41,42,43]. This fact indicates that there exists a higher risk of frailty in both persons with a higher or lower BMI [41]. Likewise, the analysis with the FTS-3 tool confirmed this fact, indicating a significant and strong association with the variable “BMI ≥ 25 kg/m^2^”. On the other hand, association studies using the FTS-3 tool indicate an association with the factor “age ≥ 75 years” with frailty, which has been described similarly in several studies, due to the intrinsic relationship with aging and the cumulative process of comorbidities [9,22,44,45]. 

## 5. Conclusions

The prevalence of frailty observed with the FTS-5 (18%) and FTS-3 (23.3%) tools are similar to the prevalence observed through the FP (19.7%) and those reported in other observational studies. Also, the results of this study provide evidence of the strong association between overweight and frail people. The limitations in this study are the size of the cohort (*n* = 300) and the presence of the pandemic and quarantine period, requiring more extensive studies to confirm the associations observed. However, considering the similar prevalence of frailty diagnosed with the three tools, the importance of screen balance dysfunction for detecting the main frailty domains affected in older people, and the simplicity and low time consumption of its application, FTS-3 may be a valuable tool for the screening of frailty in the community.

## Figures and Tables

**Table 1:** Characteristics of the studied cohort.

Variable	Women (*n* = 220)	Men (*n* = 80)	Total (*n* = 300)
Gender % (95% CI)	73.3 (68.1–78.2)	26.7 (21.9–31.9)	100
Age (mean ± SD)	74.1 ± 5.9	75.0 ± 4.9	74.3 ± 5.7
BMI (kg/m^2^) (mean ± SD)	29.7 ± 4.8	29.6 ± 3.9	29.7 ± 4.6
Years of education (mean ± SD)	9.3 ± 4.7	9.6 ± 4.0	9.4 ± 4.0
Abdominal obesity % (95% CI) ****	73.6 (67.4–79.2)	48.8 (38.1–59.6)	67.0 (61.5–72.1)
Living alone % (95% CI) *	31.4 (25.6–37.8)	18.8 (11.7–28.7)	28.0 (23.2–33.3)

* *p* < 0.05 and **** *p* < 0.0001. Continuous variables were analyzed using Mann Whitney test and categorical variables (proportions) using Pearson’s chi-squared test. BMI: body mass index and CI: confidence interval.

**Table 2:** Frequency of the frailty components of the Fried phenotype.

	Women (*n* = 220)	Men (*n* = 80)	Total (*n* = 300)
FP Components % (95% CI)			
Slowness	25.0 (19.74–31.12)	17.5 (10.72–27.26)	23.0 (18.6–28.1)
Weakness **	21.4 (16.5–27.3)	38.8 (28.8–49.7)	26.0 (21.4–31.2)
Weight loss *	16.8 (12.5–22.3)	7.5 (3.5–15.4)	14.3 (10.8–18.8)
Exhaustion	30.9 (25.2–37.3)	25.0 (16.8–35.5)	29.3 (24.5–34.7)
Low physical activity	36.4 (30.3–42.9)	33.8 (24.3–33.8)	35.7 (30.5–41.2)
Number of componentes % (95% CI)			
0	38.2 (32.0–44.8)	36.3 (26.6–47.2)	37.7 (32.4–43.3)
1	24.5 (19.3–30.6)	30.0 (21.1–40.8)	26.0 (21.4–31.2)
2	15.9 (11.7–21.3)	18.8 (11.7–28.7)	16.7 (12.9–21.3)
3	13.2 (9.4–18.3)	6.3 (13.8–2.7)	11.3 (8.2–15.4)
4	6.4 (3.8–10.4)	7.5 (3.5–15.4)	6.7 (4.4–10.1)
5	1.8 (0.7–4.6)	1.3 (0.1–6.7)	1.7 (0.7–3.8)
Total Frail (≥3 points), % (95% CI)	21.4 (16.5–27.2)	15.0 (8.8–24.4)	19.7 (15.6–24.5)
Total Pre-frail (1–2 points), % (95% CI)	40.5 (34.2–47.1)	48.8 (38.1–59.5)	42.7 (37.2–48.3)

* *p* <0.05 and ** *p* <0.001. Categorical variables (proportions) were compared using Pearson’s chi-squared test. 95% CI, 95% confidence interval; FP, Fried Phenotype.

**Table 3:** Description of the frailty criteria of the FTS-5 and FTS-3 diagnostic tools.

	Women (*n* = 220)	Men (*n* = 80)	Total (*n* = 300)
FTS-5 criteria score (mean ± SD)			
BMI	2.2 ± 2.0	1.9 ± 1.8	2.1±1.9
Handgrip ***	2.1 ± 1.9	1.3 ± 1.7	1.9 ± 1.9
Romberg Test	4.3 ± 7.4	5.8 ± 9.1	4.7 ± 7.9
Walking time **	2.4 ± 2.0	1.8 ± 1.7	2.2 ± 1.9
PASE **	4.7 ± 2.3	3.8 ± 2.6	4.4 ± 2.4
Quartiles FTS5 score % (95% CI)			
Q1	24.5 (19.3–30.6)	35 (25.5–45.9)	27.3 (22.6–32.6)
Q2	26.8 (21.4–33.0)	20 (12.7–30.0)	25.0 (20.4–30.2)
Q3	23.2 (18.1–29.2)	21.3 (13.7–31.4)	22.7 (18.3–27.7)
Q4	25.5 (20.2–31.6)	23.8 (15.8–34.1)	25.0 (20.4–30.2)
Total Frail			
FTS-5	18.1 (13.6–23.8)	17.5 (10.7–27.3)	18.0 (14.1–22.7)
FTS-3	22.7 (17.7–28.7)	25.0 (16.8–35.5)	23.3 (18.9–28.4)

** *p* < 0.01, *** *p* < 0.001. Continuous variables were analyzed using Mann Whitney test and categorical variables (proportions) using Pearson’s chi-squared test. BMI, body mass index; 95% CI; 95% confidence interval; PASE, Physical Activity Scale for the Elderly; FTS-5, Frail Trait Scale 5; FTS-3, Frail Trait Scale 3.

**Table 4:** Logistic regression for the association of frailty according to FP as a dependent variable with variables of relevance in health, adjusted by age and sex.

Variable	Frail vs. Non-Frail			Frail vs. Pre-Frail		
	Model 1 OR (95% CI)	Model 2 OR (95% CI)	Model 3 OR (95% CI)	Model 1 OR (95% CI)	Model 2 OR (95% CI)	Model 3 OR (95% CI)
Women	1.364 (0.635–0.929)	1.270 (0.561–2.875)	1.283 (0.561–2.935)	1.701 (0.812–3.564)	1.856 (0.812–4.245)	1.716 (0.738–3.989)
Age ≥ 75 years	1.434 (0.756–2.720)	1.225 (0.614–2.444)	1.264 (0.626–2.551)	0.684 (0.367–1.275)	0.700 (0.372–1.317)	0.694 (0.366–1.314)
Years of education ≤ 8		2.316 (1.171–4.579) *	2.240 (1.106–4.536) *		1.053 (0.548–2.021)	0.988 (0.509–1.919)
BMI ≥ 25 kg/m^2^		2.680 (0.663–10.838)	2.672 (0.658–10.840)		2.825 (0.705–11.325)	3.191 (0.779–13.079)
Abdominal obesity		2.113 (0.941–4.753)	2.063 (0.915–4.652)		0.811 (0.343–1.919)	0.813 (0.340–1.945)
Living alone			1.004 (0.479–2.104)			1.665 (0.808–3.432)
Automedication			0.927 (0.325–2.643)			0.642 (0.244–1.690)

OR, Odds ratio; BMI, body mass index. * *p* < 0.05.

**Table 5:** Logistic regression for the association of frailty according to FTS-5 or FTS-3 as a dependent variable with variables of relevance in health, adjusted by age and sex.

**Variable**	**FTS-5**		
	**Frail vs. Non-Frail**		
	**Model 1** **OR (95% CI)**	**Model 2** **OR (95% CI)**	**Model 3** **OR (95% CI)**
Women	1.084 (0.552–2.129)	1.010 (0.492–2.074)	0.998 (0.480–2.076)
Age ≥ 75 years	1.701 (0.937–3.087)	1.665 (0.900–3.080)	1.669 (0.900–3.096)
Years of education < 8		1.358 (0.733–2.517)	1.368 (0.735–2.548)
BMI ≥ 25 kg/m^2^		8.168 (1.031–64.676) *	7.853 (0.984–62.645)
Abdominal obesity		1.426 (0.651–3.124)	1.433 (0.650–3.160)
Living alone			0.924 (0.460–1.859)
Automedication			0.589 (0.216–1.607)
	**FTS-3**		
	**Frail vs. Non-Frail**		
	**Model 1** **OR (95% CI)**	**Model 2** **OR (95% CI)**	**Model 3** **OR (95% CI)**
Women	0.912 (0.500–1.663)	0.846 (0.444–1.613)	0.789 (0.406–1.533)
Age ≥ 75 years	1.726 (1.004–2.965) *	1.804 (1.027–3.167) *	1.839 (1.040–3.250) *
Years of education < 8		0.965 (0.547–1.702)	0.947 (0.533–1.684)
BMI ≥ 25 kg/m^2^		10.381 (1.332–80.907) *	10.225 (1.297–80.617) *
Abdominal obesity		1.664 (0.816–3.395)	1.693 (0.821–3.492)
Living alone			1.210 (0.643–2.278)
Automedication			0.390 (0.144–1.059)

OR, Odds ratio; BMI, body mass index. * *p* < 0.05.

## Data Availability

Not applicable.

## Supplementary Materials

The following supporting information can be downloaded at: https://www.mdpi.com/article/10.3390/jpm12050712/s1. Table S1: Statistical details of logistic regressions using FP as dependent variable. Table S2: Statistical details of logistic regressions using FTS-5 or FTS-3 as dependent variable.
