# Supplementary material for: Characterization by Gender of Frailty Syndrome in Elderly People according to Frail Trait Scale and Fried Frailty Phenotype
Source: J Pers Med. 2022 Apr 29;12(5):712. doi: 10.3390/jpm12050712 (PMC9144746; doi:10.3390/jpm12050712)
Supplement: Supplementary file 1 [file jpm-12-00712-s001.zip › jpm-1674834-supplementary.pdf]

SUPPLEMENTARY DATA

Statistical details of logistic regressions

The statistical analyses performed in each logistic regression are detailed below.

Table S1. Statistical details of logistic regressions using FP as dependent variable.

Variable	Frail vs Non-frail											
	Model 1				Model 2				Model 3			
	S.E	Wald statistic	d.f	p-value	S.E	Wald statistic	d.f	p-value	S.E	Wald statistic	d.f	p-value
Women	0.354	1.473	1	0.225	0.373	0.910	1	0.340	0.377	0.707	1	0.401
Age ≥ 75 years	0.293	0.008	1	0.930	0.302	0.106	1	0.745	0.303	0.090	1	0.764
Years of education ≤ 8					0.303	3.916	1	0.028	0.305	3.710	1	0.035
BMI ≥ 25					0.667	2.334	1	0.127	0.669	2.377	1	0.123
Abdominal obesity					0.383	0.628	1	0.643	0.384	0.638	1	0.424
Living alone									0.325	0.457	1	0.499
Automedication									0.449	0.167	1	0.683
**Variable**	**Frail vs Non-frail**											
	**Model 1**				**Model 2**				**Model 3**			
	**S.E**	**Wald statistic**	**d.f**	**p-value**	**S.E**	**Wald statistic**	**d.f**	**p-value**	**S.E**	**Wald statistic**	**d.f**	**p-value**
Women	0.377	1.979	1	0.159	0.422	2.148	1	0.143	0.428	1.764	1	0.184
Age ≥ 75 years	0.318	1.429	1	0.232	0.323	1.225	1	0.268	0.325	1.255	1	0.263
Years of education ≤ 8					0.333	0.024	1	0.878	0.338	0.001	1	0.973
BMI ≥ 25					0.708	2.149	1	0.143	0.718	2.621	1	0.105
Abdominal obesity					0.439	0.227	1	0.634	0.443	0.187	1	0.666
Living alone									0.366	1.672	1	0.196
Automedication									0.482	0.593	1	0.441

This table is related to Table 4 "Logistic regression for the association of frailty according to FP as a dependent variable with variables of relevance in health, adjusted by age and sex". S.E, standard error; d.f, degree of freedom.

Table S2. Statistical details of logistic regressions using FTS-5 or FTS-3 as dependent variable.

Variable	Frail vs Non-frail (according to FTS-5)											
	Model 1				Model 2				Model 3			
	S.E	Wald statistic	d.f	p-value	S.E	Wald statistic	d.f	p-value	S.E	Wald statistic	d.f	p-value
Women	0.345	0.054	1	0.816	0.367	0.001	1	0.979	0.374	0.0001	1	0.996
Age ≥ 75 years	0.304	3.049	1	0.081	0.314	2.634	1	0.105	0.315	2.642	1	0.104
Years of education ≤ 8					0.315	0.944	1	0.331	0.317	0.976	1	0.323
BMI ≥ 25					1.056	3.957	1	0.047	1.060	3.784	1	0.052
Abdominal obesity					0.400	0.785	1	0.376	0.403	0.795	1	0.372
Living alone									0.357	0.049	1	0.825
Automedication									0.512	1.069	1	0.301
**Variable**	**Frail vs Non-frail (according to FTS-5)**											
	**Model 1**				**Model 2**				**Model 3**			
	**S.E**	**Wald statistic**	**d.f**	**p-value**	**S.E**	**Wald statistic**	**d.f**	**p-value**	**S.E**	**Wald statistic**	**d.f**	**p-value**
Women	0.307	0.091	1	0.763	0.329	0.258	1	0.611	0.339	0.487	1	0.485
Age ≥ 75 years	0.276	3.904	1	0.048	0.287	4.216	1	0.040	0.291	4.396	1	0.036
Years of education ≤ 8					0.290	0.015	1	0.901	0.294	0.034	1	0.854
BMI ≥ 25					1.048	4.989	1	0.026	1.054	4.870	1	0.027
Abdominal obesity					0.364	1.961	1	0.161	0.370	2.029	1	0.154
Living alone									0.323	0.349	1	0.555
Automedication									0.510	3.415	1	0.065

This table is related to Table 5 "Logistic regression for the association of frailty according to FTS-5 or FTS-3 as a dependent variable with variables of relevance in health, adjusted by age and sex". S.E, standard error; d.f, degree of freedom.
